# Supplementary material for: YM155-Adapted Cancer Cell Lines Reveal Drug-Induced Heterogeneity and Enable the Identification of Biomarker Candidates for the Acquired Resistance Setting
Source: Cancers (Basel). 2020 Apr 26;12(5):1080. doi: 10.3390/cancers12051080 (PMC7281096; doi:10.3390/cancers12051080)
Supplement: Supplementary file 1 [file cancers-12-01080-s001.pdf]

Table S1. YM155 concentrations (nM) that reduce the viability of UKF-NB-3 or YM155-adapted UKF-NB-3 sub-lines by 50% (IC_{50}) or 90% (IC_{90}) as indicated by MTT assay after 120h of incubation and doubling times of the cells.

	IC_{50}	IC_{90}	Doubling times (h)
UKF-NB-3	0.55 ± 0.06	1.01 ± 0.24	30.8 ± 0.8
UKF-NB-3rYM155^{20nM}I	36.2 ± 2.0 (66)[1]	94.8 ± 0.7 (94)[2]	41.5 ± 5.2
UKF-NB-3rYM155^{20nM}II	23.6 ± 2.2 (43)	39.9 ± 0.9 (40)	39.2 ± 4.8
UKF-NB-3rYM155^{20nM}III	31.8 ± 2.3 (58)	49.1 ± 0.2 (49)	32.3 ± 1.1
UKF-NB-3rYM155^{20nM}IV	21.0 ± 0.6 (38)	29.8 ± 4.8 (30)	32.2 ± 2.0
UKF-NB-3rYM155^{20nM}V	25.1 ± 0.1 (45)	39.5 ± 0.7 (39)	35.3 ± 2.8
UKF-NB-3rYM155^{20nM}VI	41.9 ± 5.3 (76)	136 ± 7 (135)	36.3 ± 2.2
UKF-NB-3rYM155^{20nM}VII	36.5 ± 5.5 (66)	96.2 ± 23.9 (95)	46.1 ± 2.1
UKF-NB-3rYM155^{20nM}VIII	34.5 ± 0.6 (63)	84.7 ± 27.3 (84)	47.0 ± 2.5
UKF-NB-3rYM155^{20nM}IX	27.2 ± 0.5 (49)	44.8 ± 2.9 (44)	33.3 ± 0.2
UKF-NB-3rYM155^{20nM}X	26.9 ± 0.5 (49)	45.2 ± 4.7 (45)	41.9 ± 2.2

[1] fold resistance (IC_{50} YM155-adapted sub-line/ IC_{50} UKF-NB-3)
[2] fold resistance (IC_{90} YM155-adapted sub-line/ IC_{90} UKF-NB-3)

Table S2. Drug concentrations that reduce the viability of UKF-NB-3 or YM155-adapted UKF-NB-3 sub-lines by 50% (IC_{50}) as indicated by MTT assay after 120h of incubation.

	Nutlin-3	Vincristine	Cisplatin	Gemcitabine	Topotecan
	IC_{50} (μM)	IC_{50} (ng/mL)	IC_{50} (ng/mL)	IC_{50} (ng/mL)	IC_{50} (ng/mL)
UKF-NB-3	1.05 ± 0.25	1.75 ± 0.55	169 ± 29	0.30 ± 0.03	1.29 ± 0.52
UKF-NB-3rYM155^{20nM}I	0.57 ± 0.07 (0.5)[1]	45.5 ± 11.1 (26)	157 ± 54 (0.9)	0.64 ± 0.02 (2.1)	1.37 ± 0.53 (1.1)
UKF-NB-3rYM155^{20nM}II	1.31 ± 0.03 (1.2)	27.0 ± 12.6 (15)	183 ± 51 (1.1)	0.50 ± 0.04 (1.7)	1.25 ± 0.53 (1.0)
UKF-NB-3rYM155^{20nM}III	1.27 ± 0.01 (1.2)	10.8 ± 6.4 (6.2)	122 ± 24 (0.7)	0.62 ± 0.01 (2.1)	1.06 ± 0.24 (0.8)
UKF-NB-3rYM155^{20nM}IV	0.47 ± 0.03 (0.4)	18.5 ± 8.4 (11)	159 ± 38 (0.9)	0.23 ± 0.04 (0.8)	1.56 ± 0.65 (1.2)
UKF-NB-3rYM155^{20nM}V	0.99 ± 0.13 (0.9)	8.90 ± 7.39 (5.1)	156 ± 84 (0.9)	0.12 ± 0.04 (0.4)	0.91 ± 0.41 (0.7)
UKF-NB-3rYM155^{20nM}VI	0.64 ± 0.01 (0.6)	714 ± 456 (408)	132 ± 39 (0.8)	0.64 ± 0.01 (2.1)	1.55 ± 0.72 (1.2)
UKF-NB-3rYM155^{20nM}VII	1.27 ± 0.04 (1.2)	28.8 ± 10.2 (16)	134 ± 6 (0.8)	0.19 ± 0.01 (0.6)	1.44 ± 0.84 (1.1)
UKF-NB-3rYM155^{20nM}VIII	0.70 ± 0.01 (0.7)	39.5 ± 15.4 (23)	190 ± 56 (1.1)	0.65 ± 0.01 (2.2)	1.26 ± 0.50 (1.0)
UKF-NB-3rYM155^{20nM}IX	0.33 ± 0.01 (0.3)	5.63 ± 1.94 (3.2)	178 ± 41 (1.1)	0.18 ± 0.01 (0.6)	1.59 ± 0.74 (1.2)
UKF-NB-3rYM155^{20nM}X	0.64 ± 0.15 (0.6)	26.0 ± 6.2 (15)	144 ± 44 (0.9)	0.54 ± 0.01 (1.8)	1.21 ± 0.40 (0.9)

[1] fold resistance (IC_{50} YM155-adapted sub-line/ IC_{50} UKF-NB-3)

Figure S1

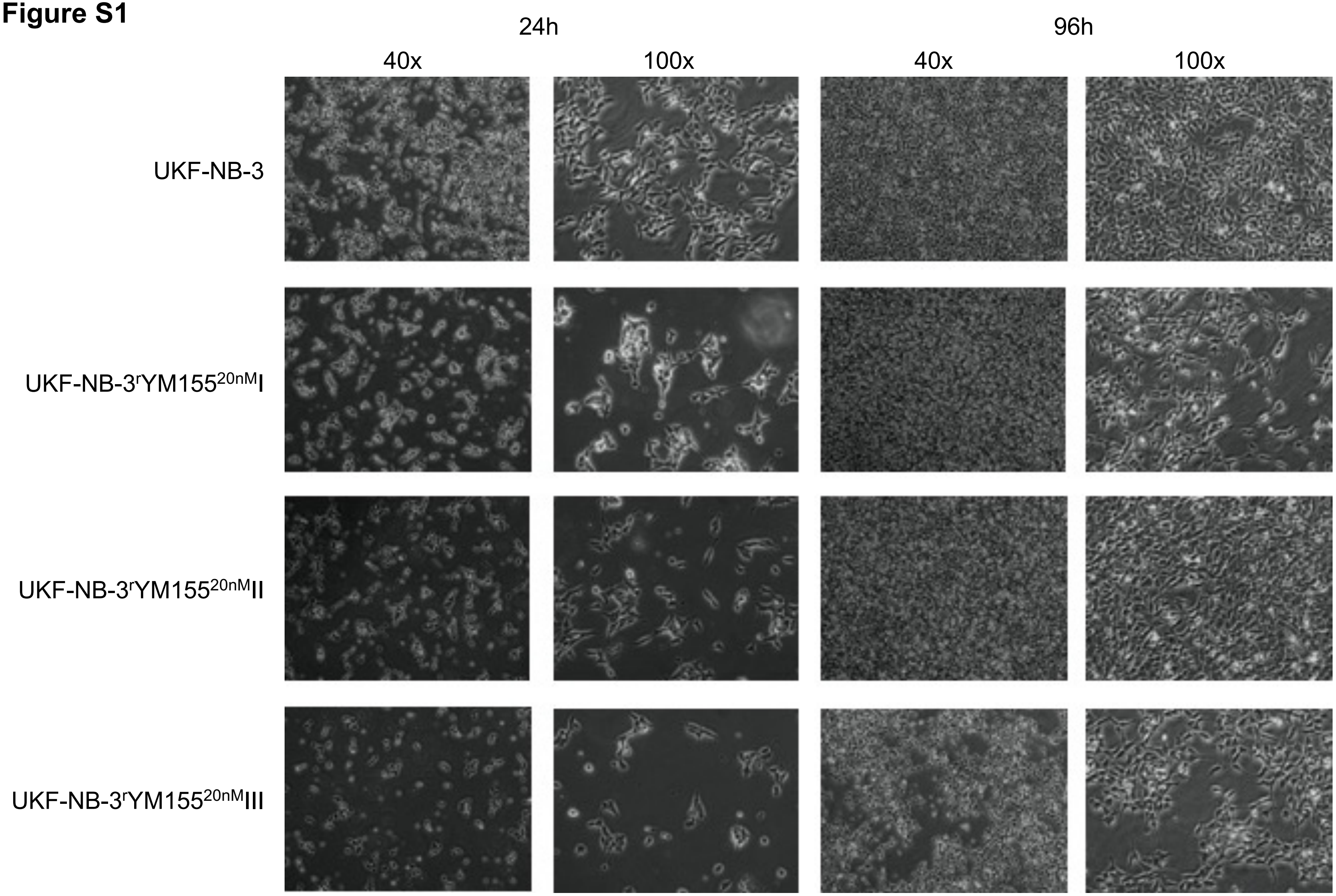
24h
96h
40x
100x
40x
100x
UKF-NB-3
UKF-NB-3^{r}YM155^{20nM}I
UKF-NB-3^{r}YM155^{20nM}II
UKF-NB-3^{r}YM155^{20nM}III

Figure S1

24h 96h

40x 100x 40x 100x

UKF-NB-3^{r}YM155^{20nM}IV

UKF-NB-3^{r}YM155^{20nM}V

UKF-NB-3^{r}YM155^{20nM}VI

UKF-NB-3^{r}YM155^{20nM}VII

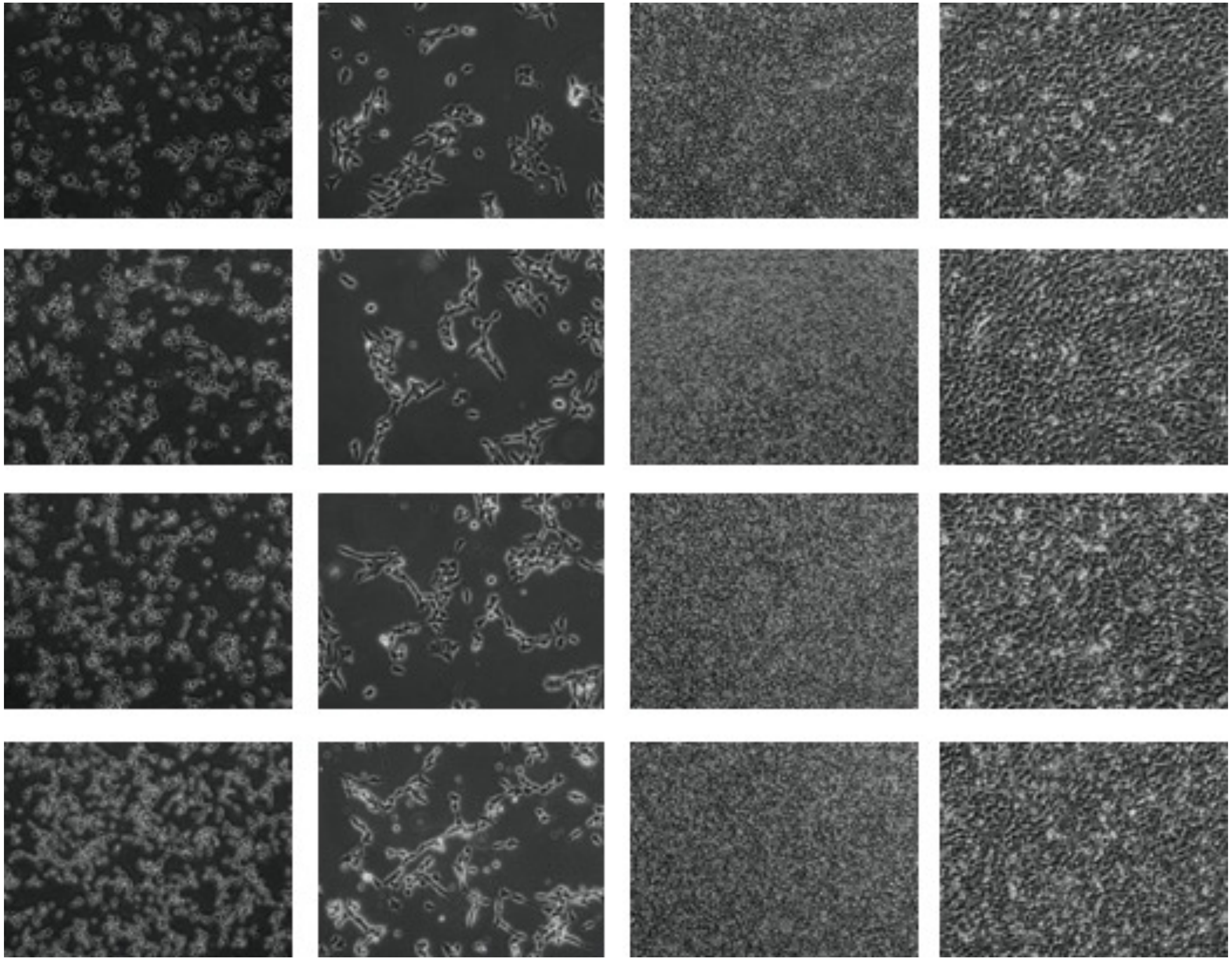

Figure S1

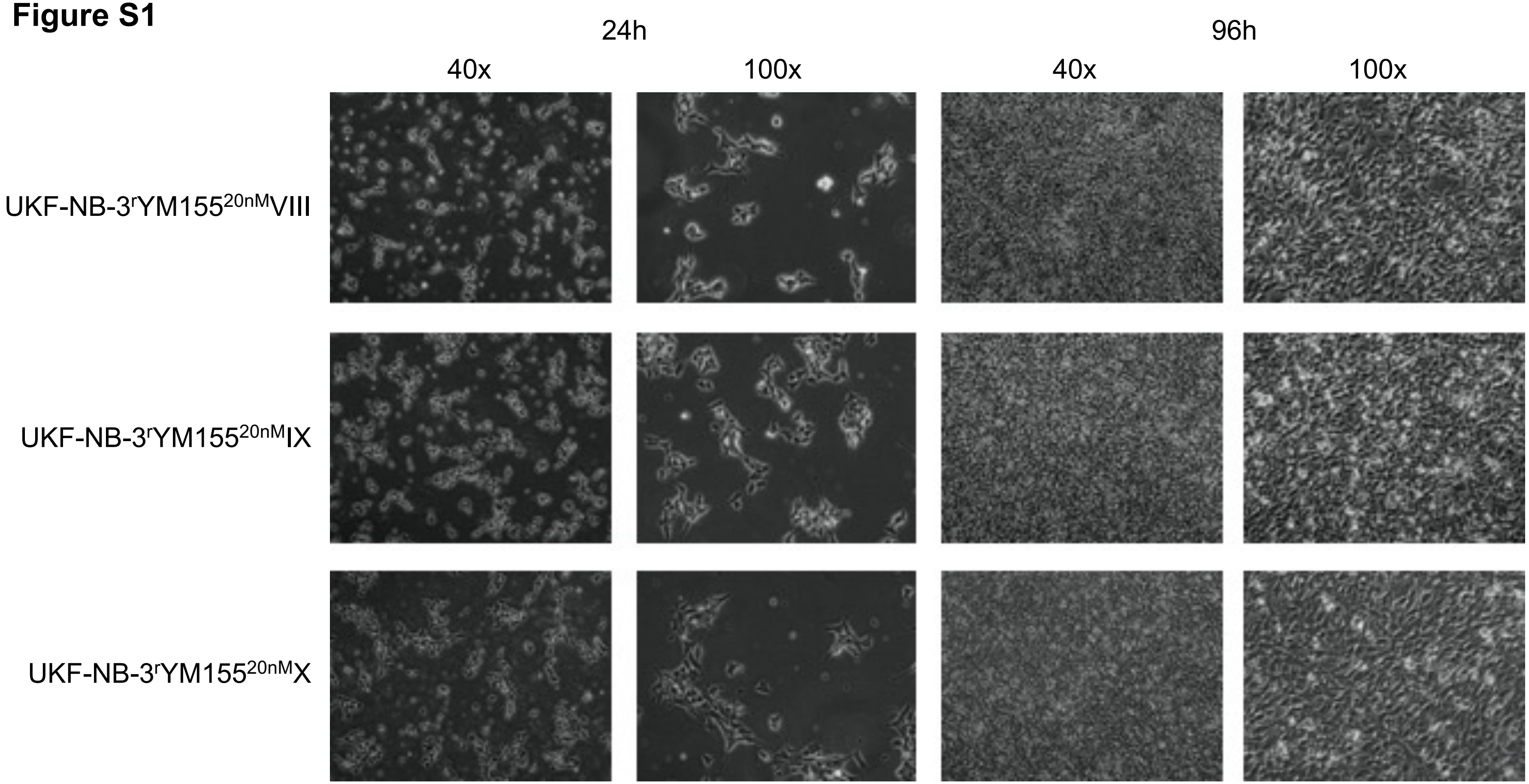

Figure S1. Representative photos of the project cell lines indicating cell morphology after different periods of cultivation and at different maginifications.

Figure S2

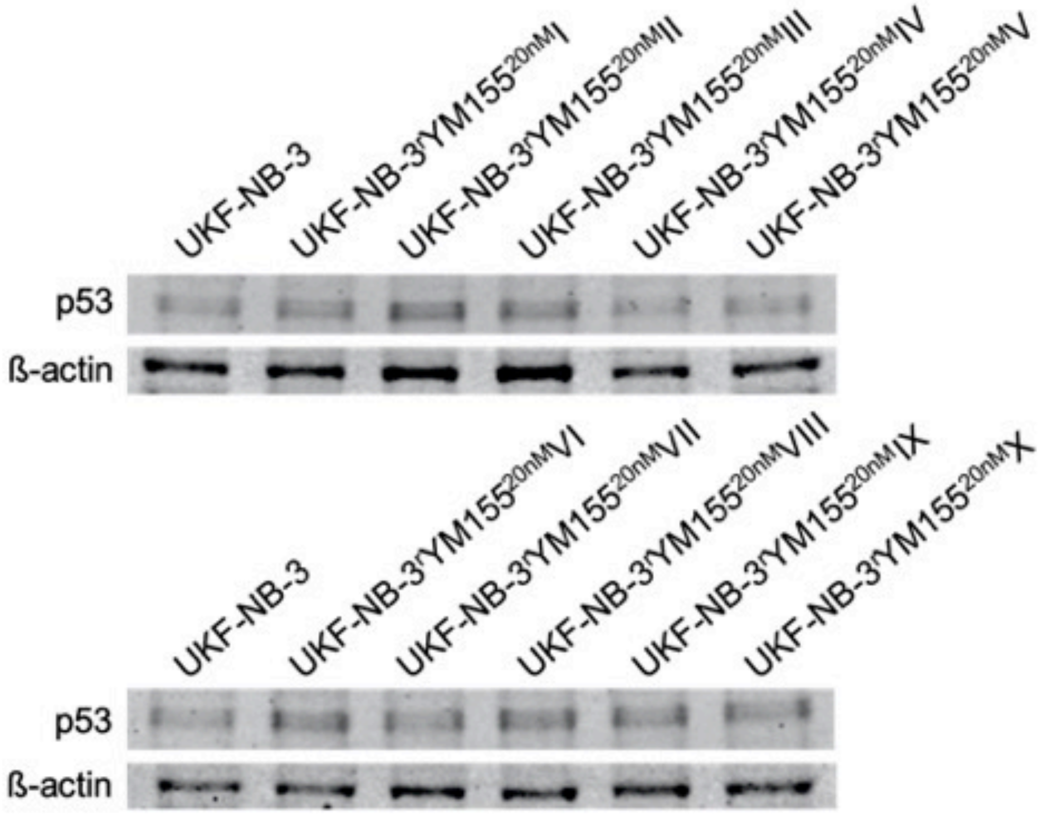

Figure S2. p53 levels in UKF-NB-3 and its YM155-adapted sublines.

Figure S2. Representative Western blots indicating cellular levels of p53 in UKF-NB-3 and YM155-adapted UKF-NB-3 sub-lines. Densitometric analysis was performed with QuantiOne (BioRad). p53 levels were normalised to β-actin expression and values relative to control cells are displayed.

Figure S2

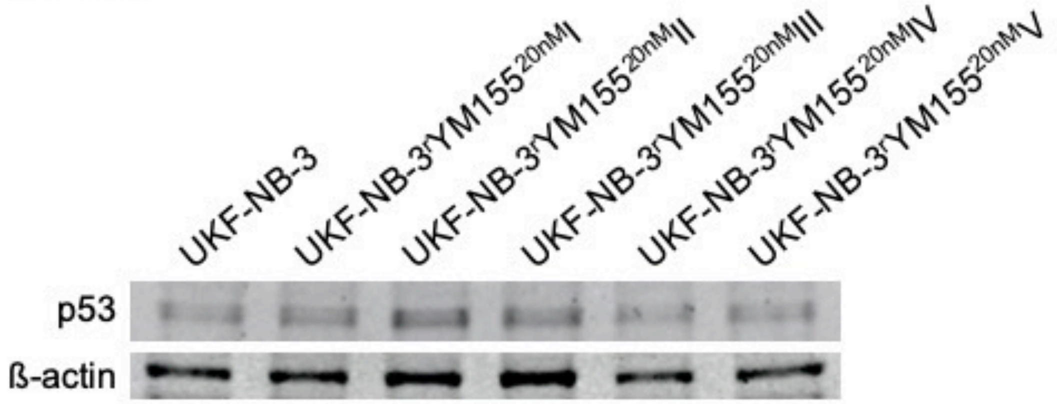
UKF-NB-3
UKF-NB-3rYM155 20nM I
UKF-NB-3rYM155 20nM II
UKF-NB-3rYM155 20nM III
UKF-NB-3rYM155 20nM IV
UKF-NB-3rYM155 20nM V
p53
ß-actin

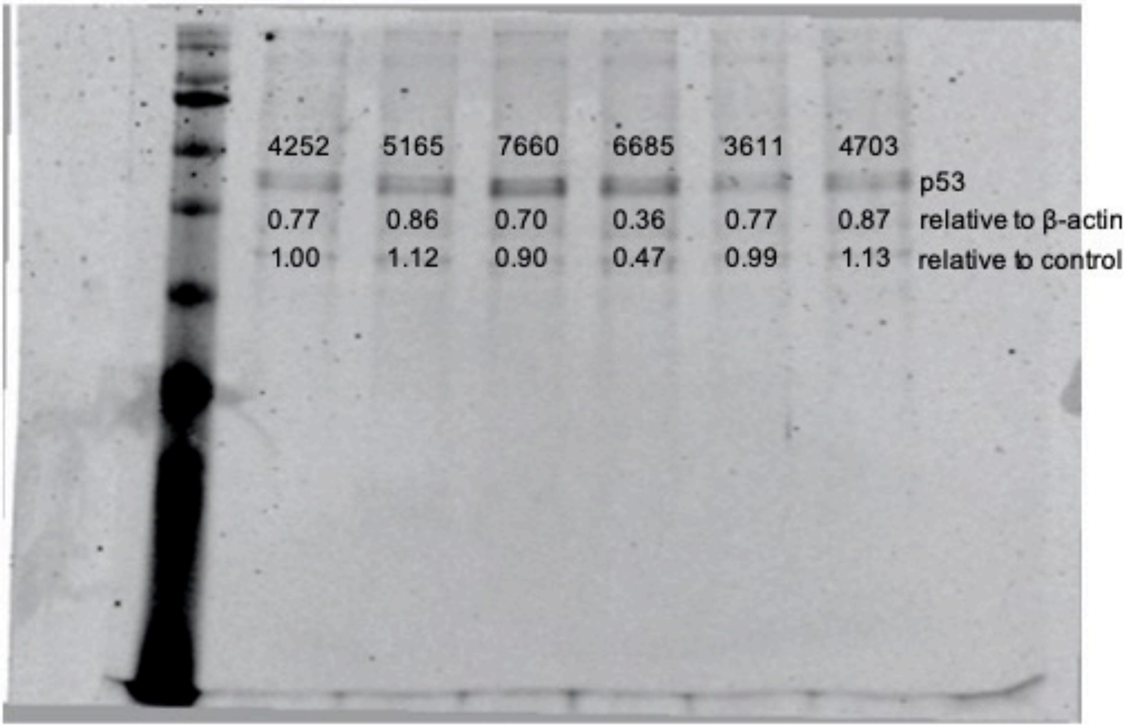
4252
5165
7660
6685
3611
4703
p53
0.77
0.86
0.70
0.36
0.77
0.87
relative to β-actin
1.00
1.12
0.90
0.47
0.99
1.13
relative to control

Figure S2

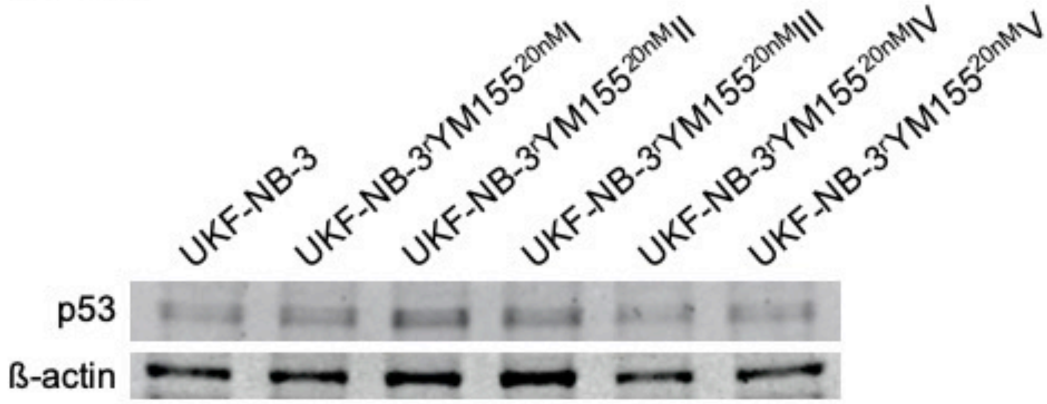
UKF-NB-3
UKF-NB-3rYM155 20nM I
UKF-NB-3rYM155 20nM II
UKF-NB-3rYM155 20nM III
UKF-NB-3rYM155 20nM IV
UKF-NB-3rYM155 20nM V
p53
ß-actin

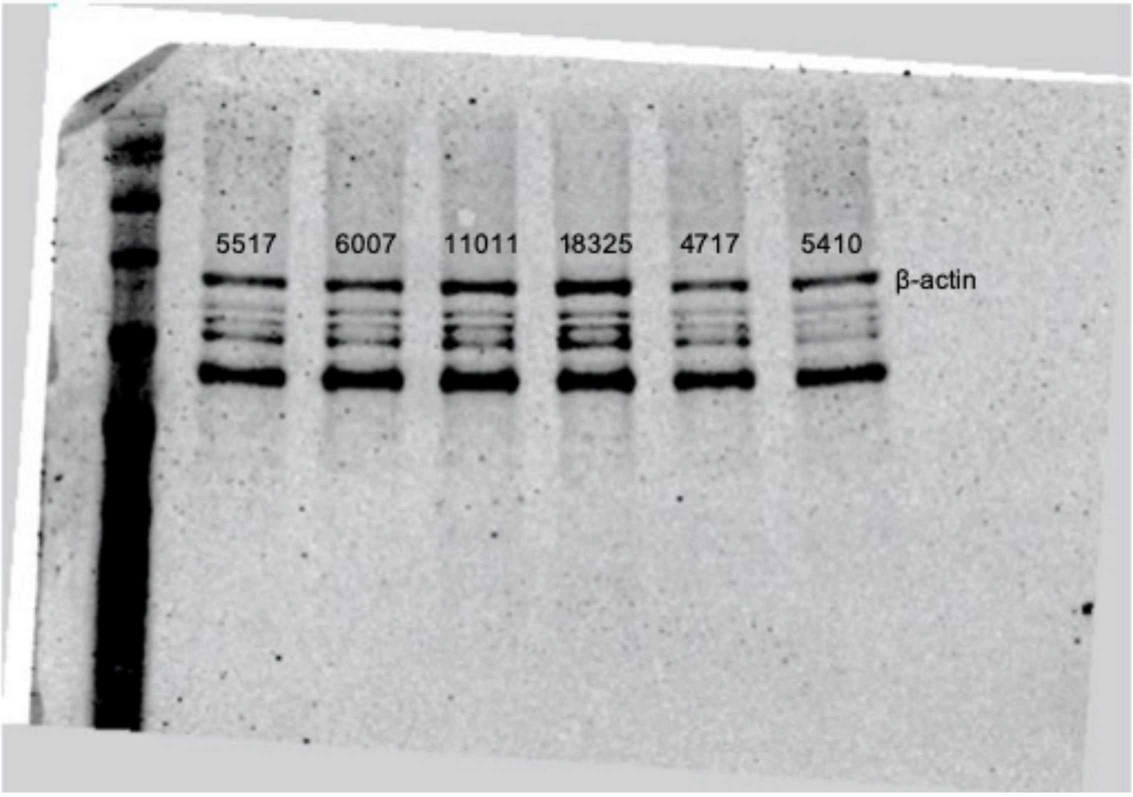
5517
6007
11011
18325
4717
5410
β-actin

Figure S2

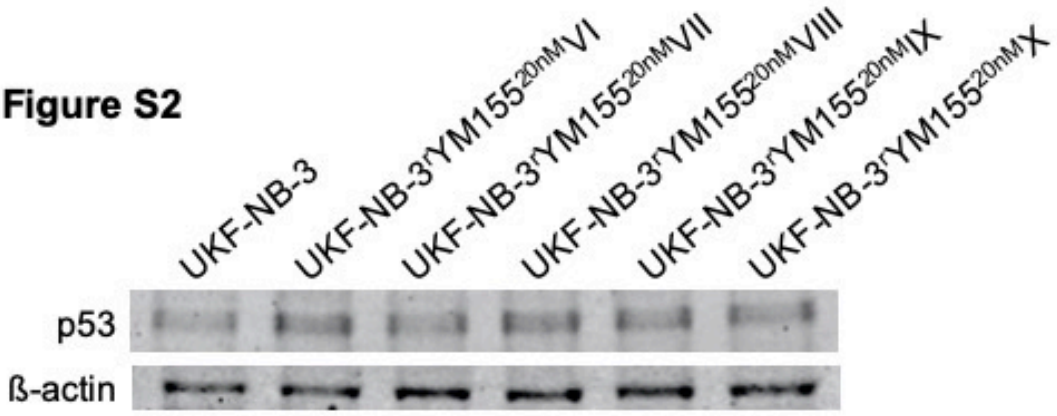

UKF-NB-3
UKF-NB-3^{r}YM155^{20nM}VI
UKF-NB-3^{r}YM155^{20nM}VII
UKF-NB-3^{r}YM155^{20nM}VIII
UKF-NB-3^{r}YM155^{20nM}IX
UKF-NB-3^{r}YM155^{20nM}X
p53
ß-actin

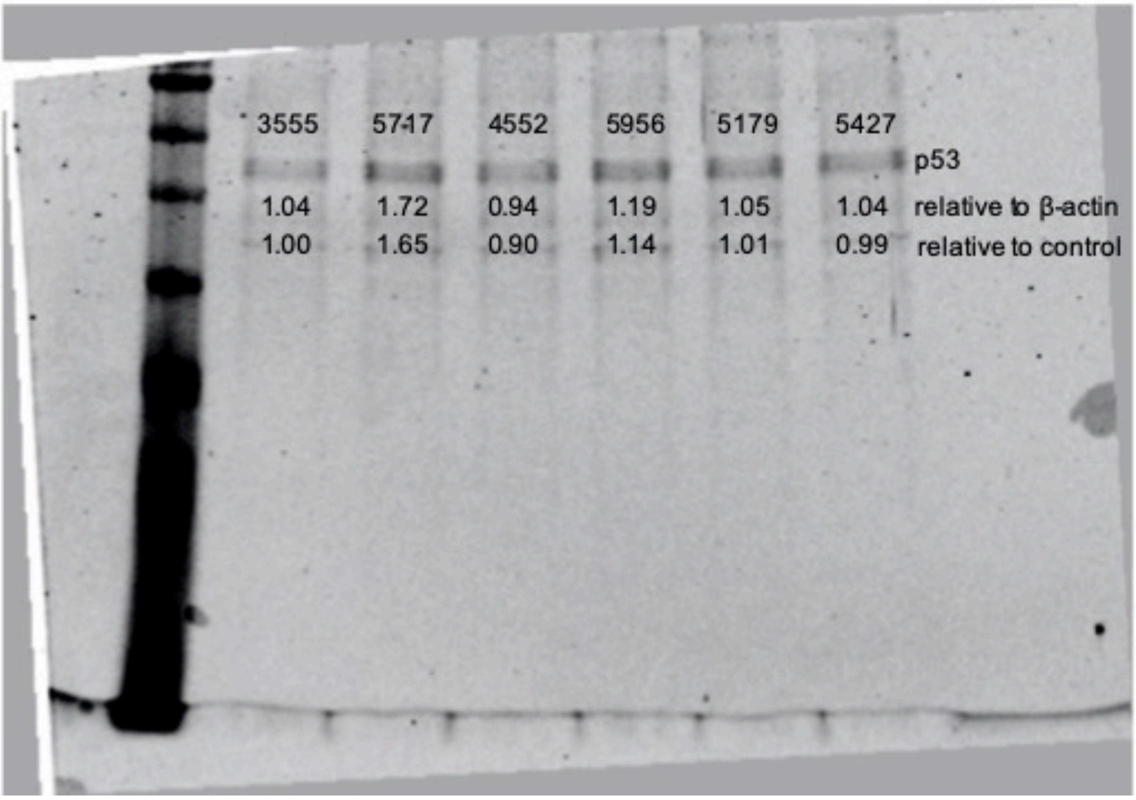

3555 5717 4552 5956 5179 5427
p53
1.04 1.72 0.94 1.19 1.05 1.04 relative to β-actin
1.00 1.65 0.90 1.14 1.01 0.99 relative to control

Figure S2

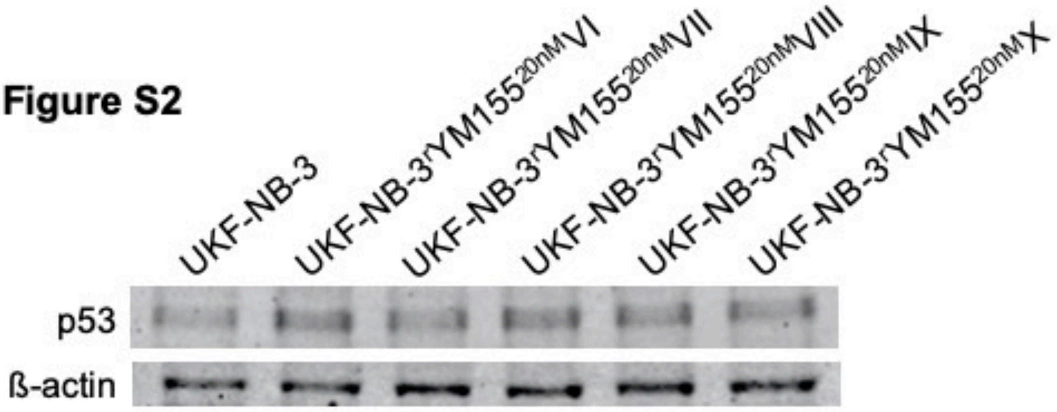
UKF-NB-3
UKF-NB-3^{r}YM155^{20nM}VI
UKF-NB-3^{r}YM155^{20nM}VII
UKF-NB-3^{r}YM155^{20nM}VIII
UKF-NB-3^{r}YM155^{20nM}IX
UKF-NB-3^{r}YM155^{20nM}X
p53
ß-actin

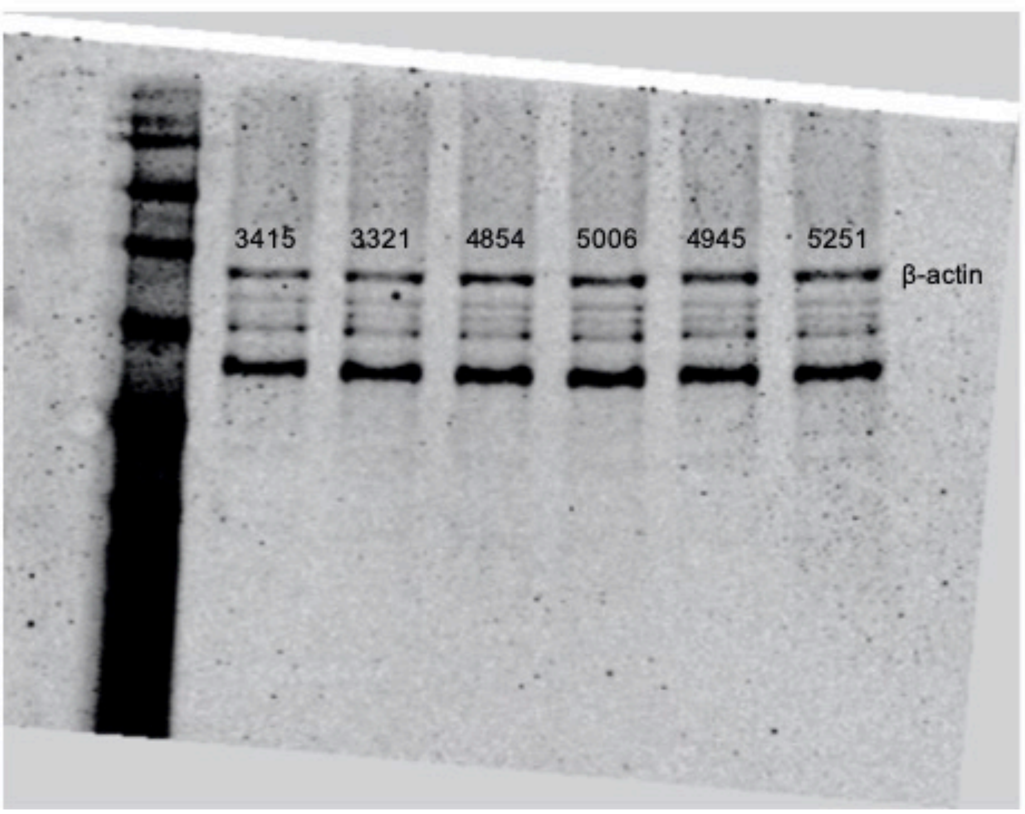
3415
3321
4854
5006
4945
5251
β-actin

Figure S3

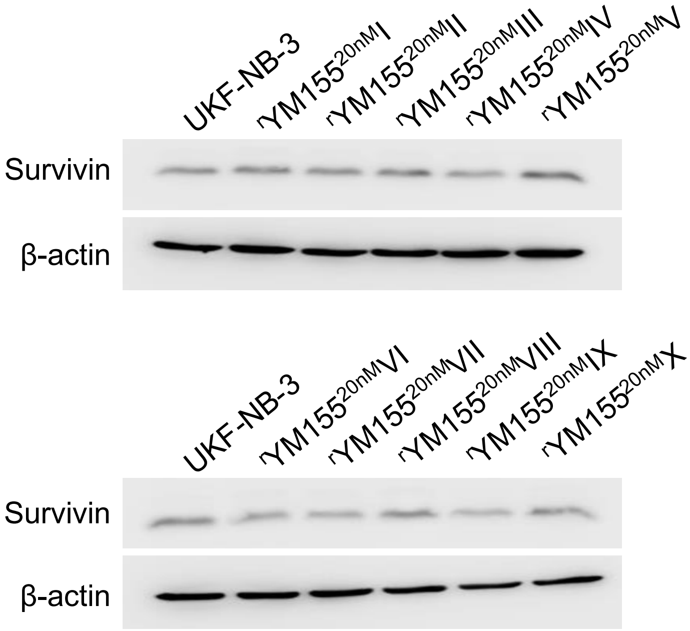

Figure S3. Representative Western blots indicating cellular levels of survivin in UKF-NB-3 and YM155-adapted UKF-NB-3 sub-lines. Densitometric analysis was performed with Image Studio Ver. 5.2 software (LICOR). Survivin levels were normalised to β-actin expression and values relative to control cells are displayed.

Figure S3

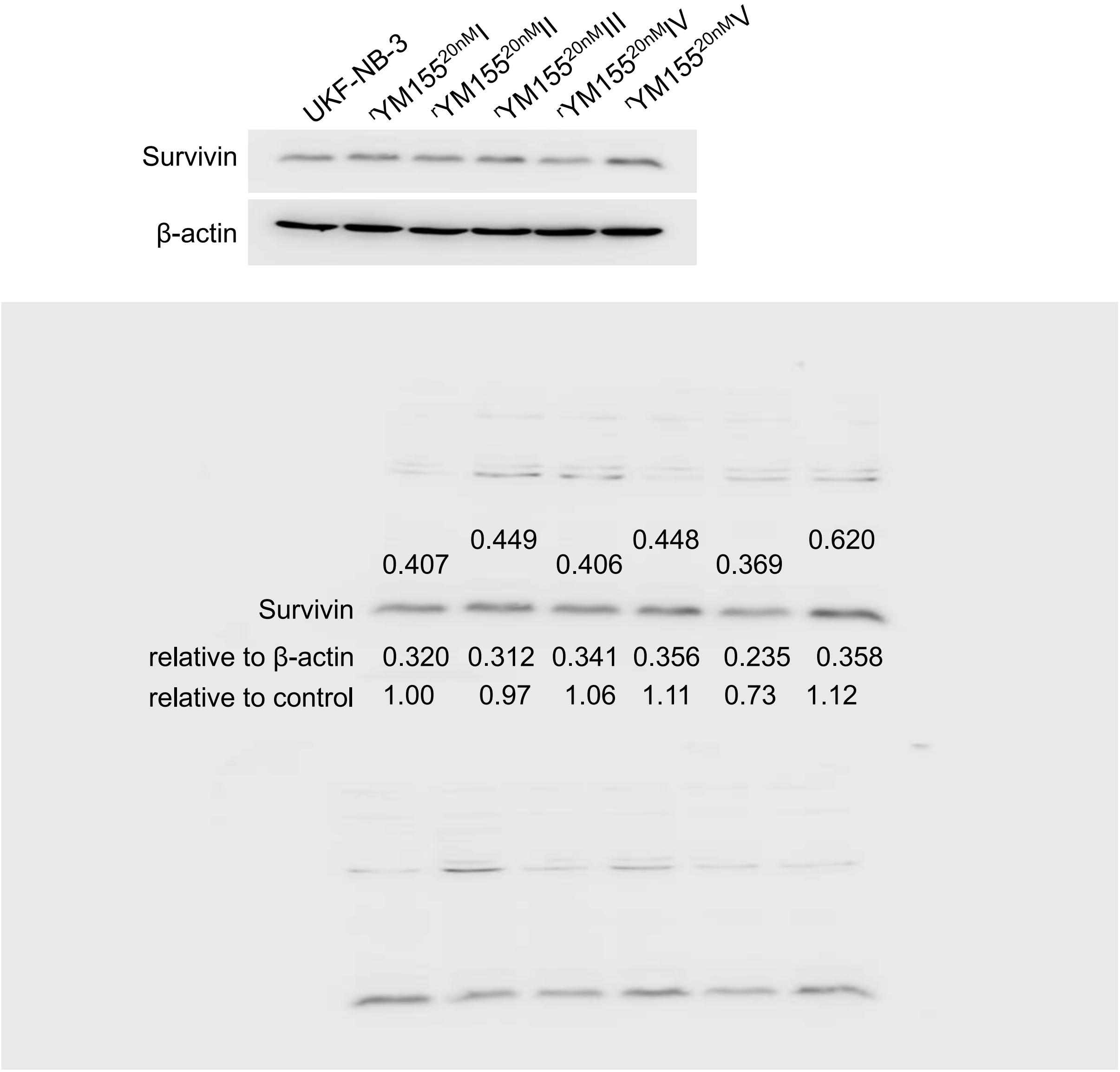
UKF-NB-3
rYM155 20nM I
rYM155 20nM II
rYM155 20nM III
rYM155 20nM IV
rYM155 20nM V
Survivin
β-actin
0.407
0.449
0.406
0.448
0.369
0.620
Survivin
relative to β-actin 0.320 0.312 0.341 0.356 0.235 0.358
relative to control 1.00 0.97 1.06 1.11 0.73 1.12

Figure S3

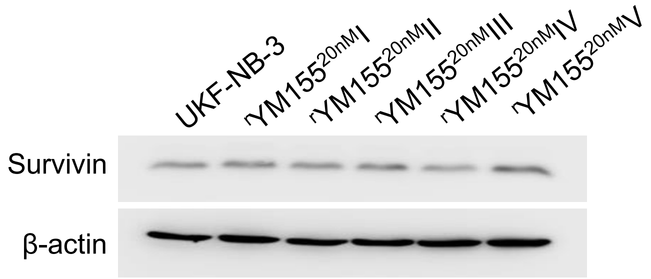
UKF-NB-3
rYM155 20nM I
rYM155 20nM II
rYM155 20nM III
rYM155 20nM IV
rYM155 20nM V
Survivin
β-actin

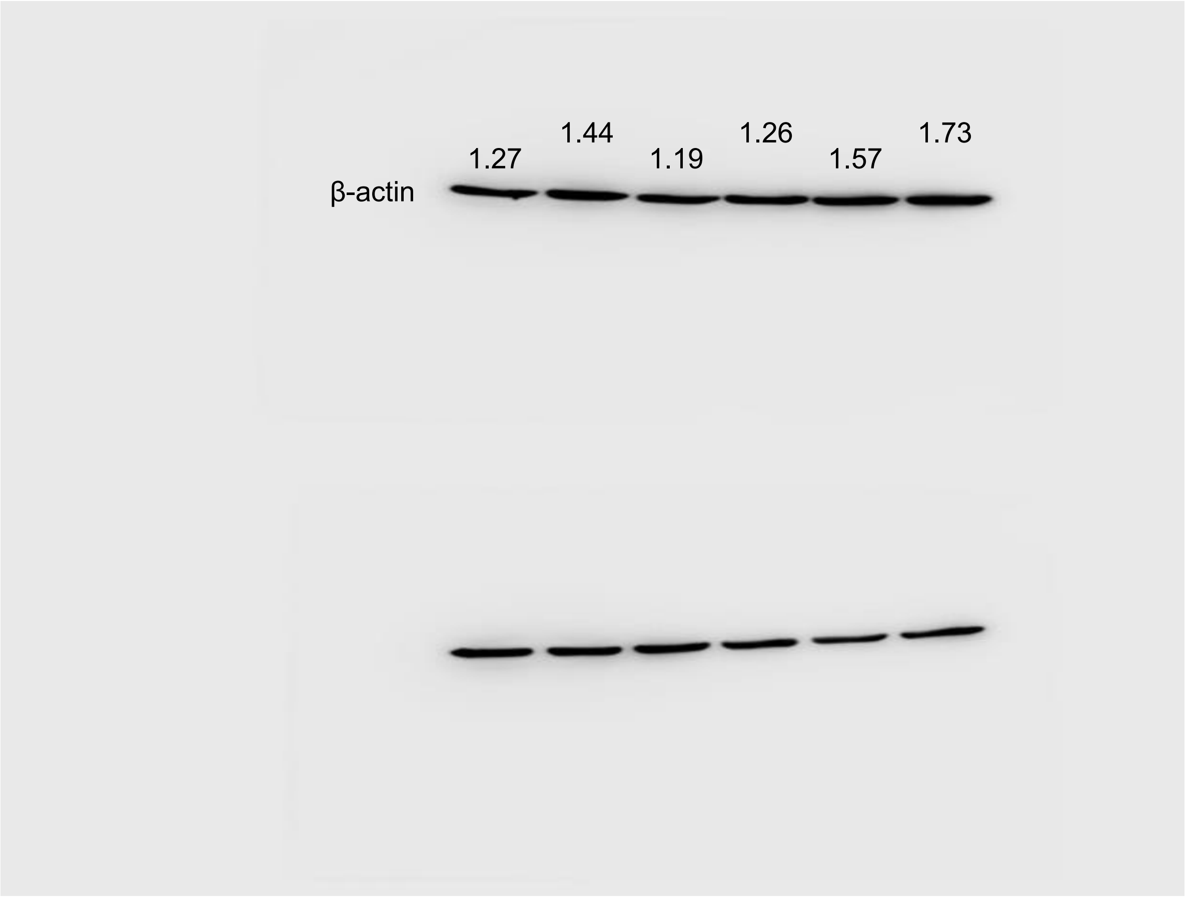
1.27
1.44
1.19
1.26
1.57
1.73
β-actin

Figure S3

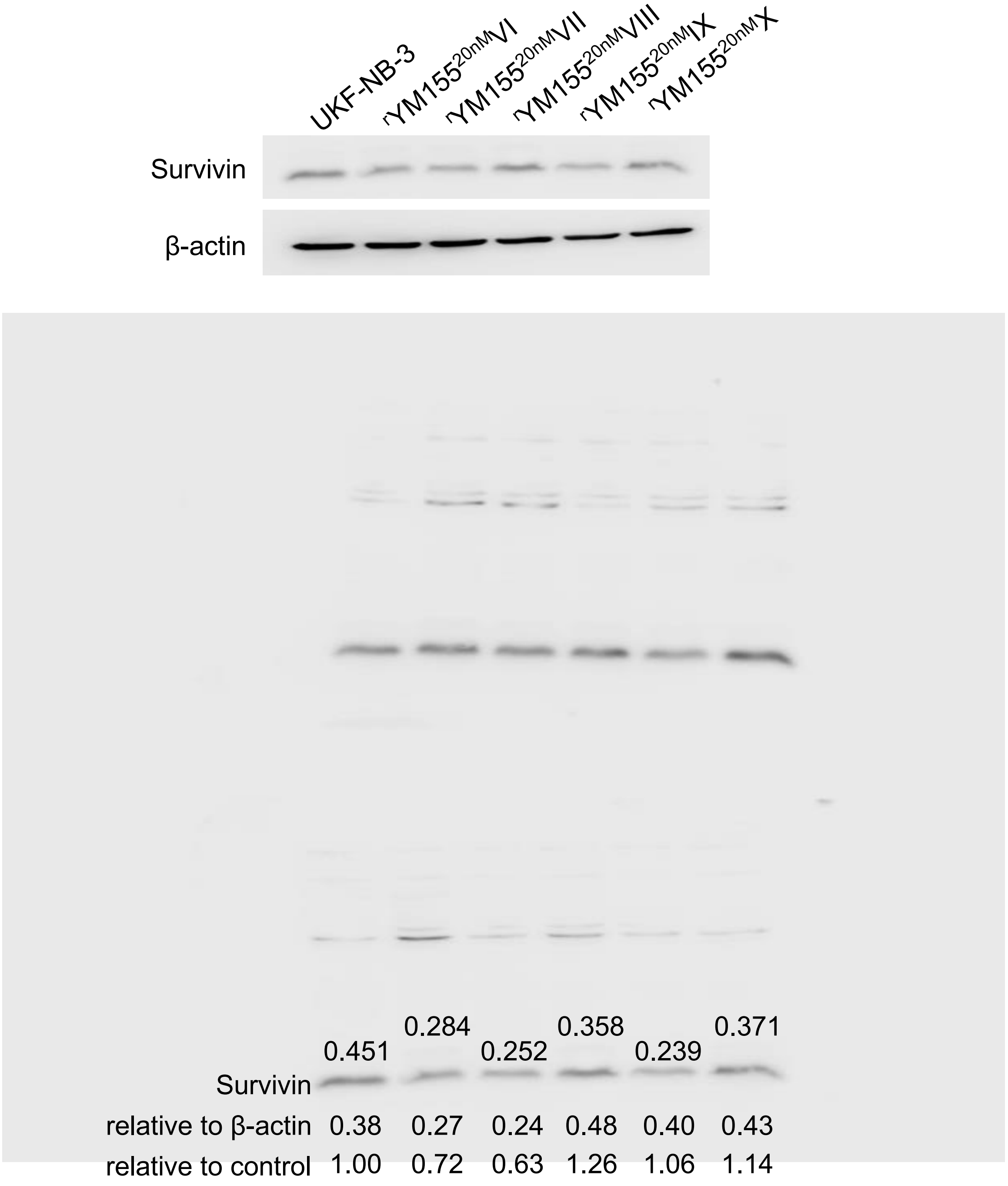
UKF-NB-3
rYM155 20nMVI
rYM155 20nMVII
rYM155 20nMVIII
rYM155 20nMIX
rYM155 20nMX
Survivin
β-actin
0.451
0.284
0.252
0.358
0.239
0.371
Survivin
relative to β-actin 0.38 0.27 0.24 0.48 0.40 0.43
relative to control 1.00 0.72 0.63 1.26 1.06 1.14

Figure S3

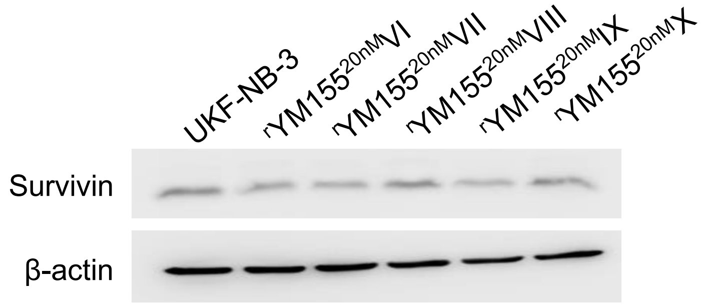
UKF-NB-3
rYM155 20nM VI
rYM155 20nM VII
rYM155 20nM VIII
rYM155 20nM IX
rYM155 20nM X
Survivin
β-actin

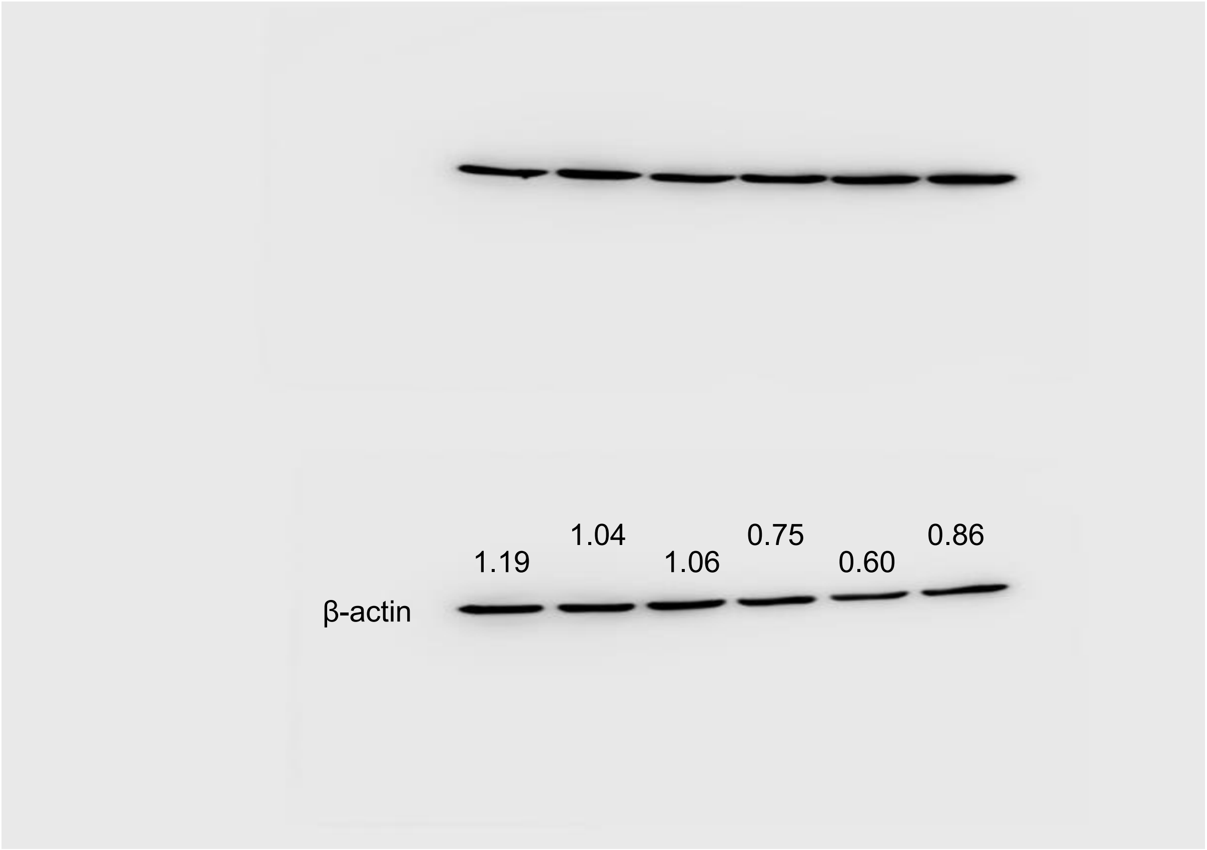
1.19
1.04
1.06
0.75
0.60
0.86
β-actin

Figure S4
survivin

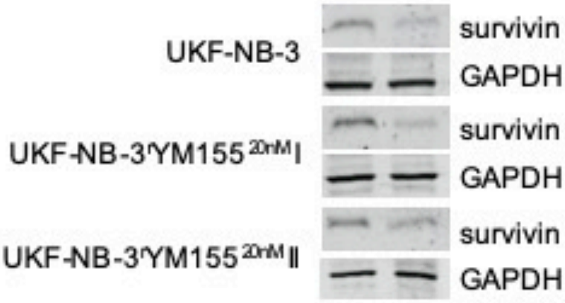

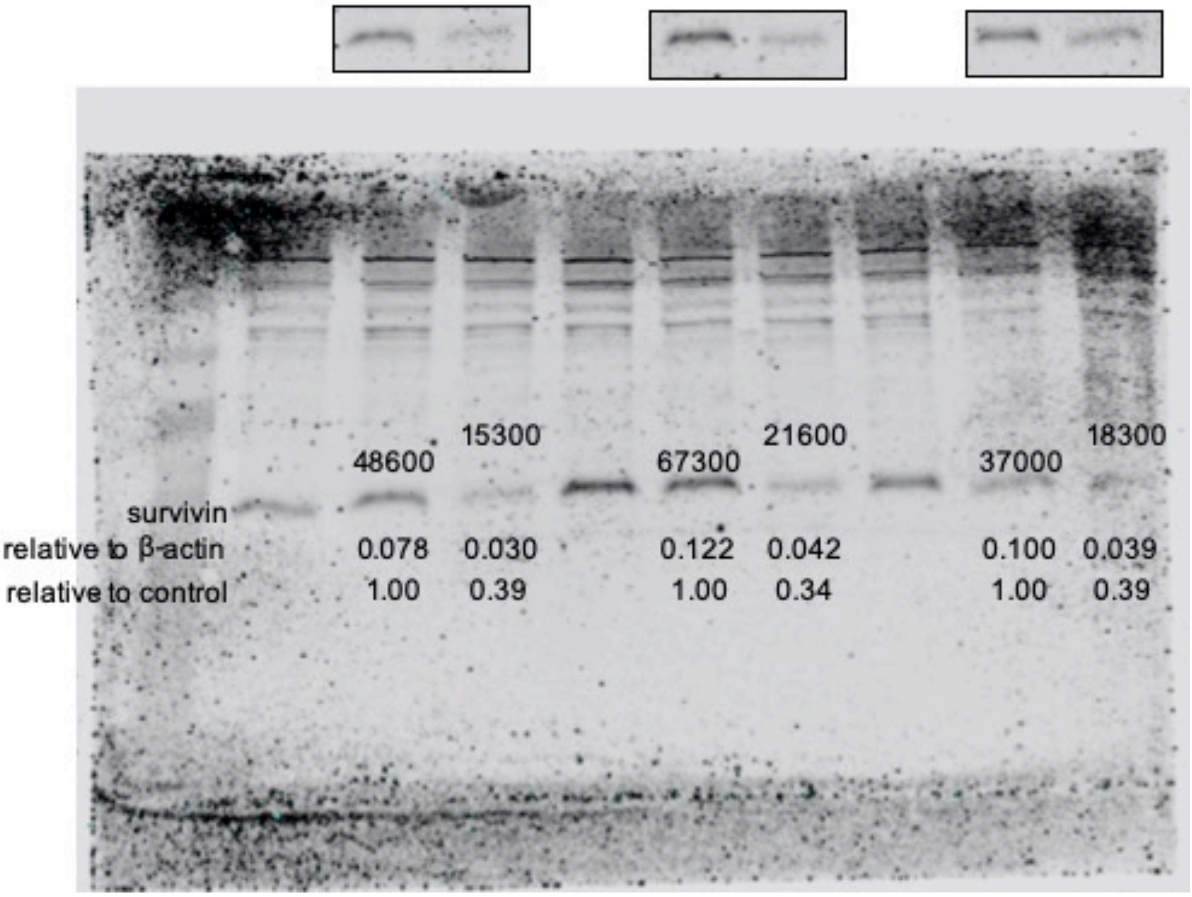

Figure S4
GAPDH

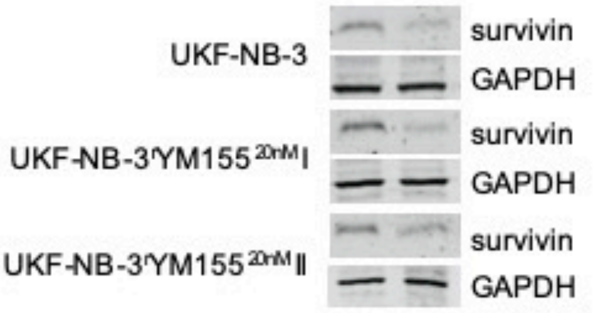

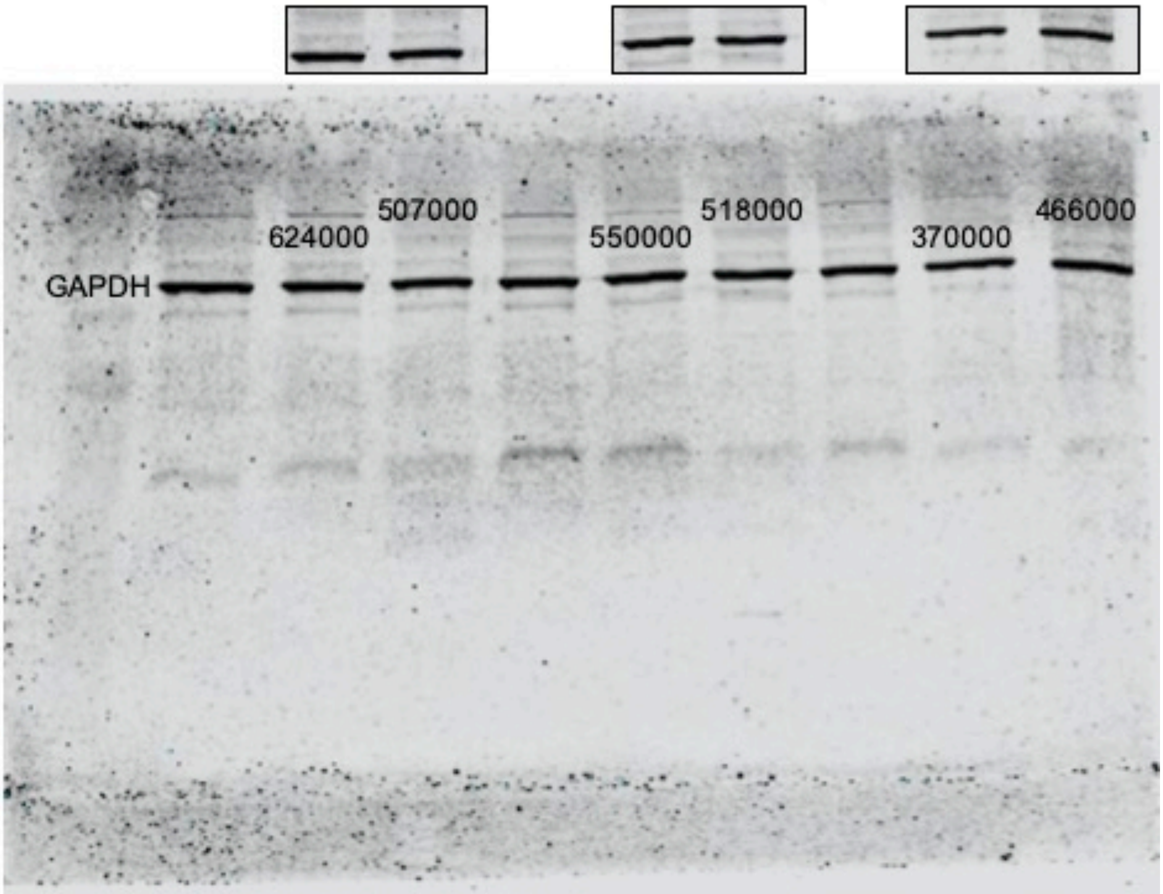

Figure S4
survivin

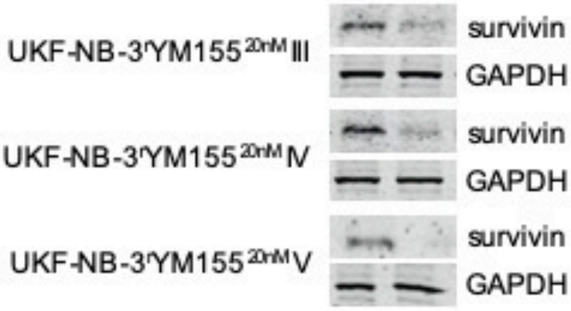

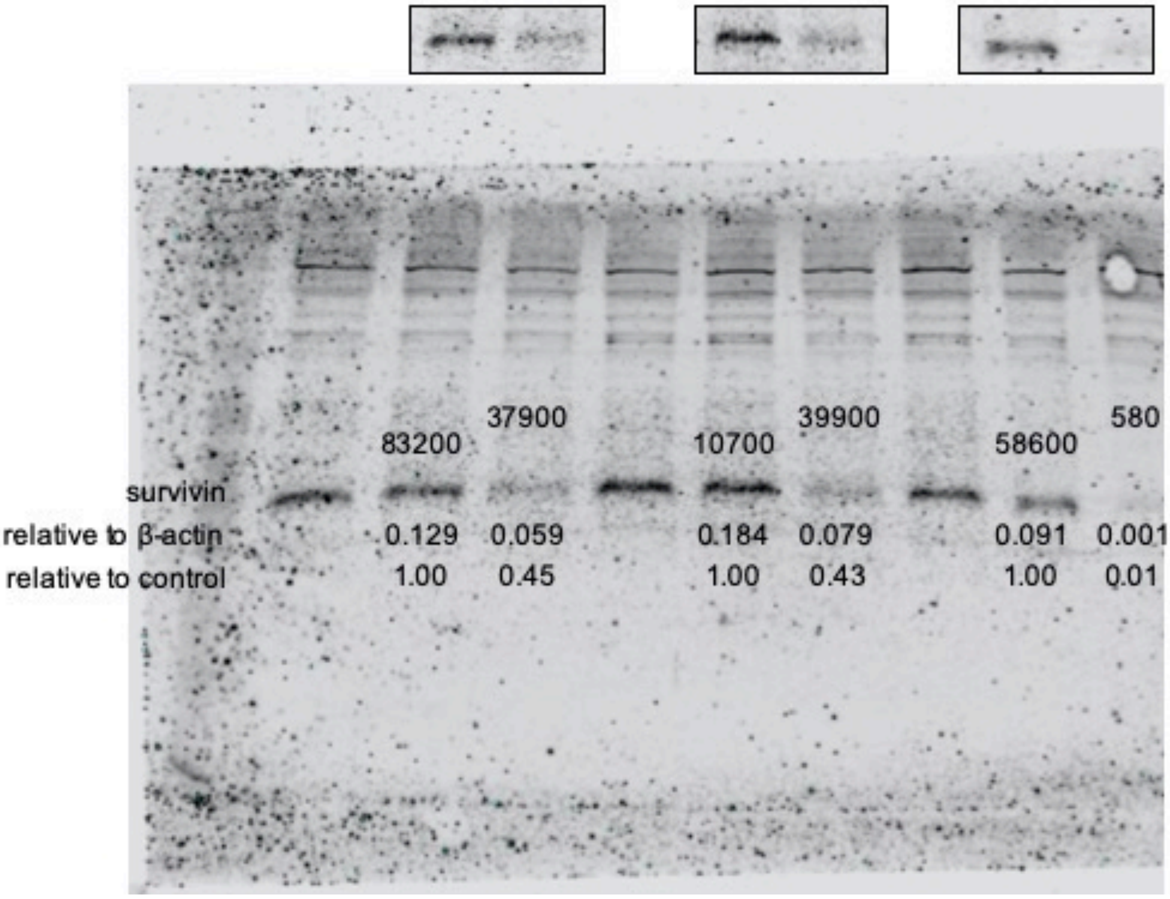

Figure S4

GAPDH

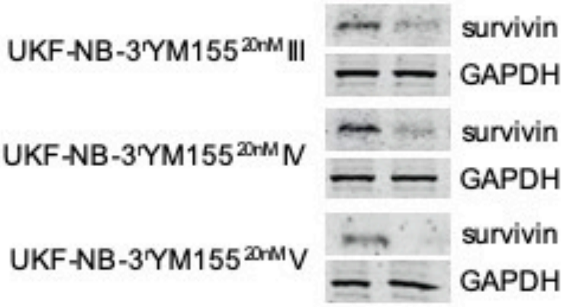

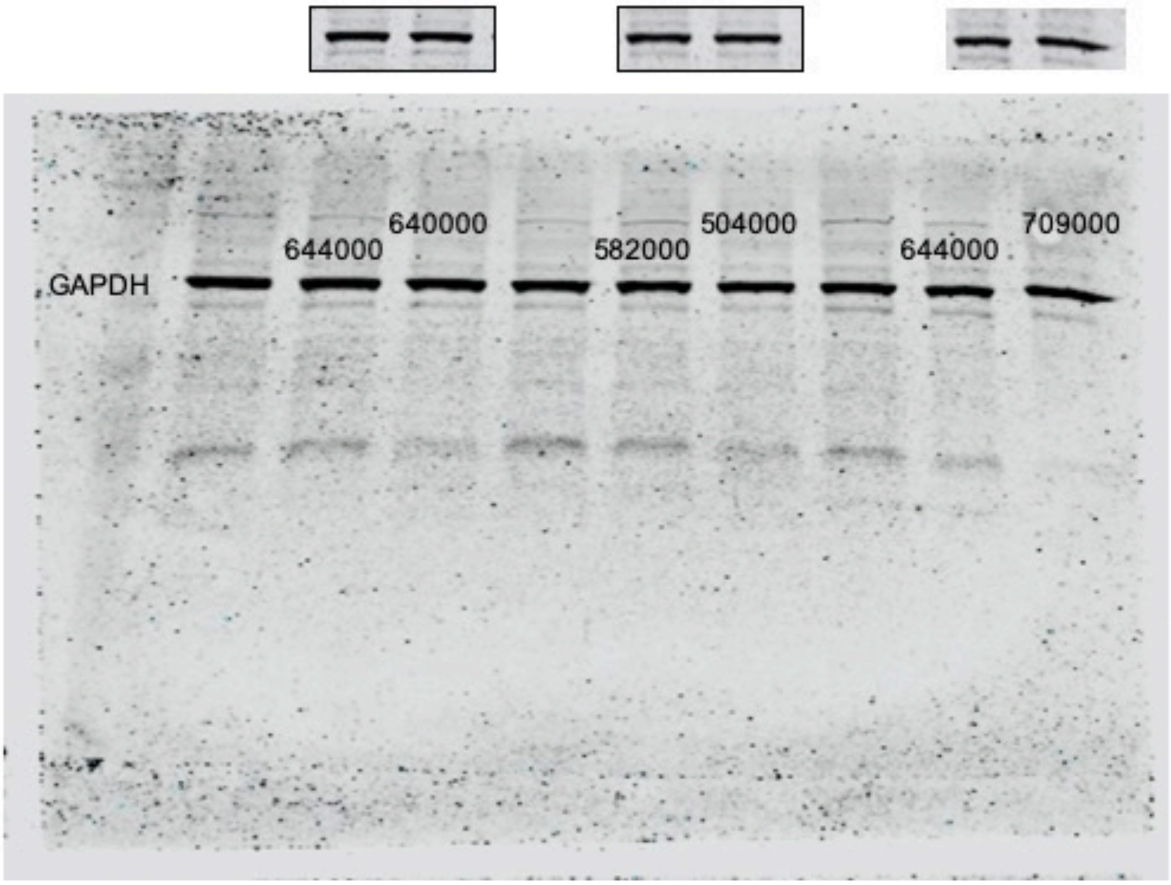

Figure S4
survivin

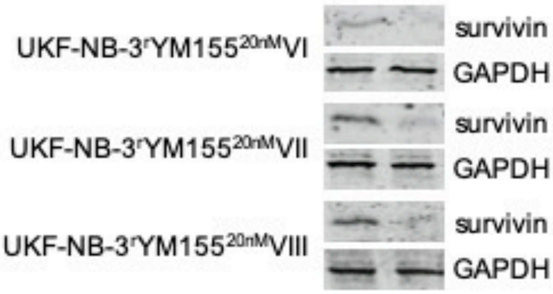

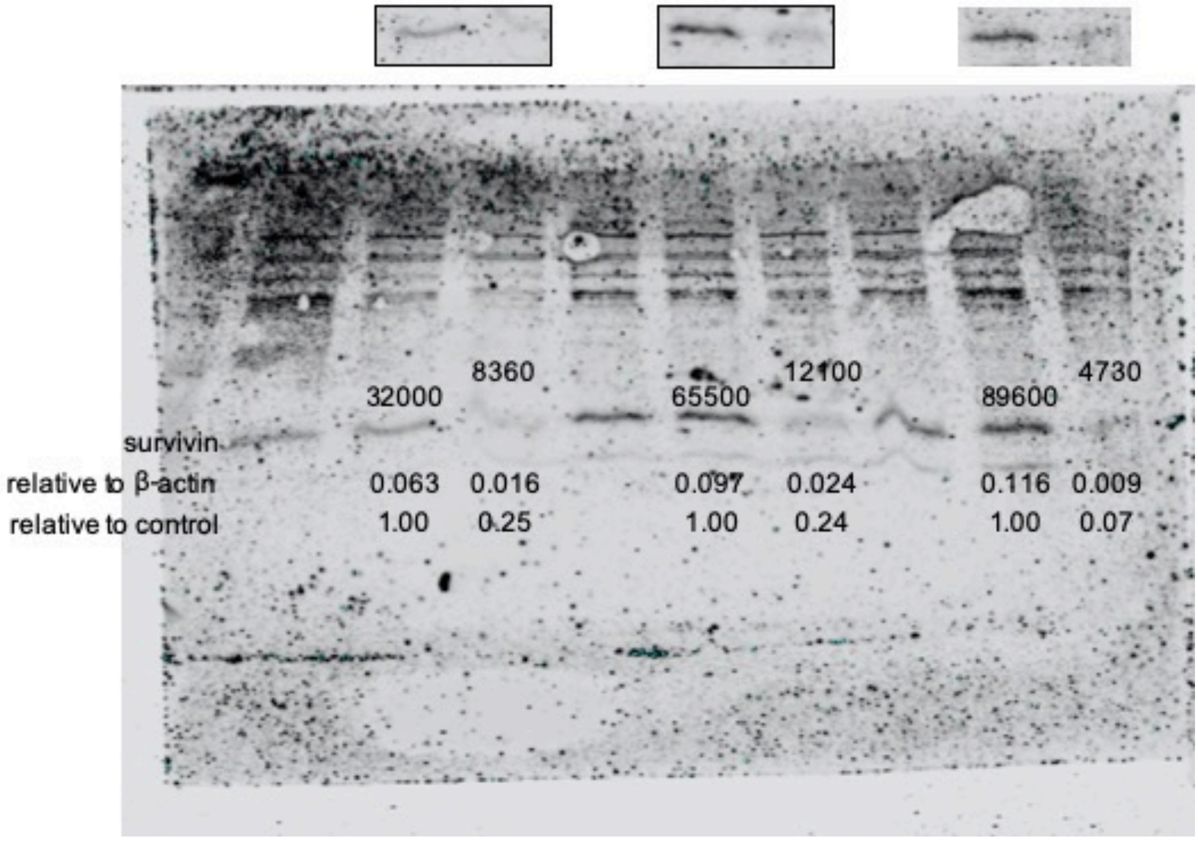

Figure S4
GAPDH

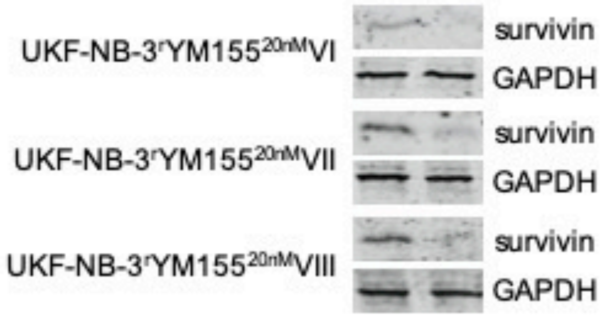

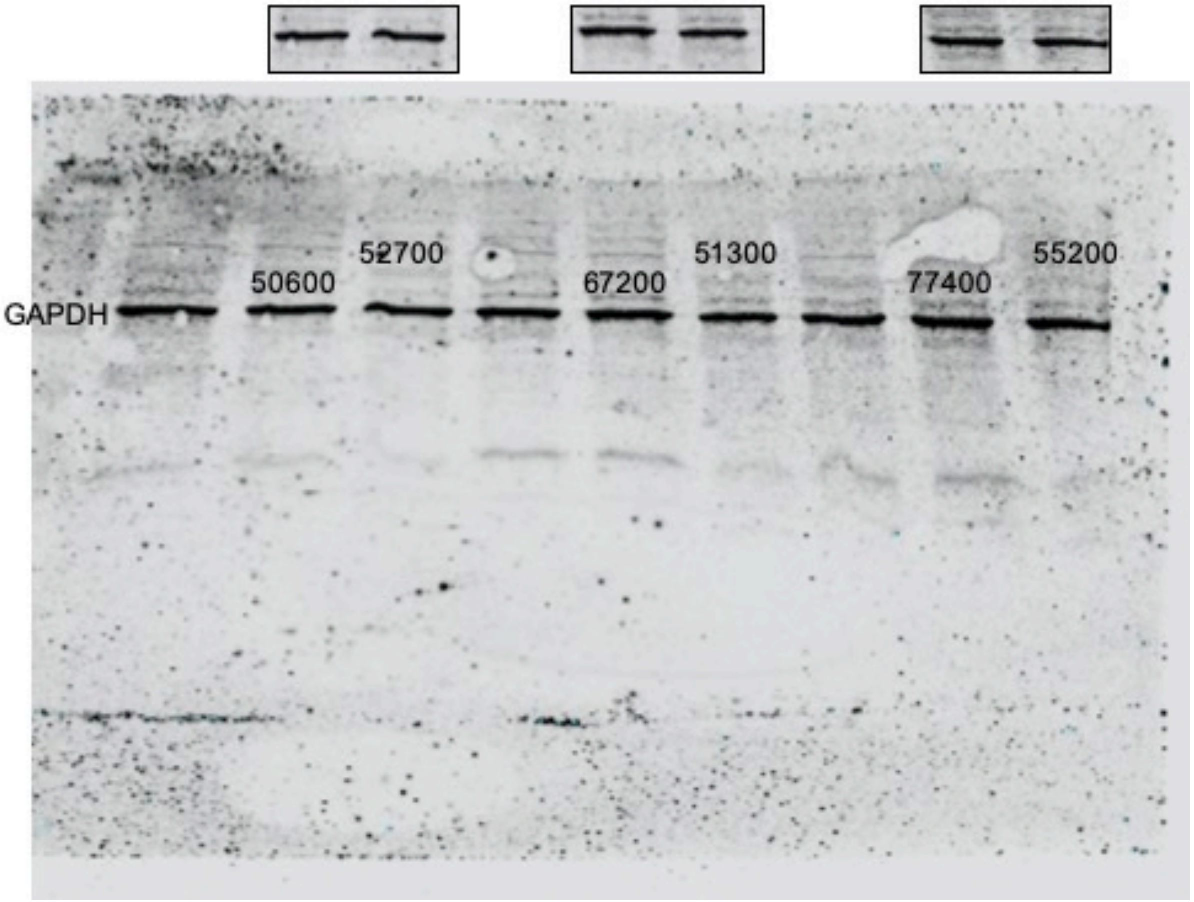

Figure S4
survivin

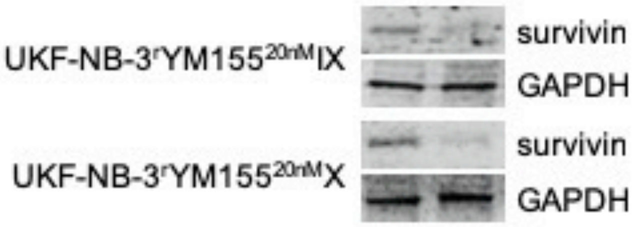

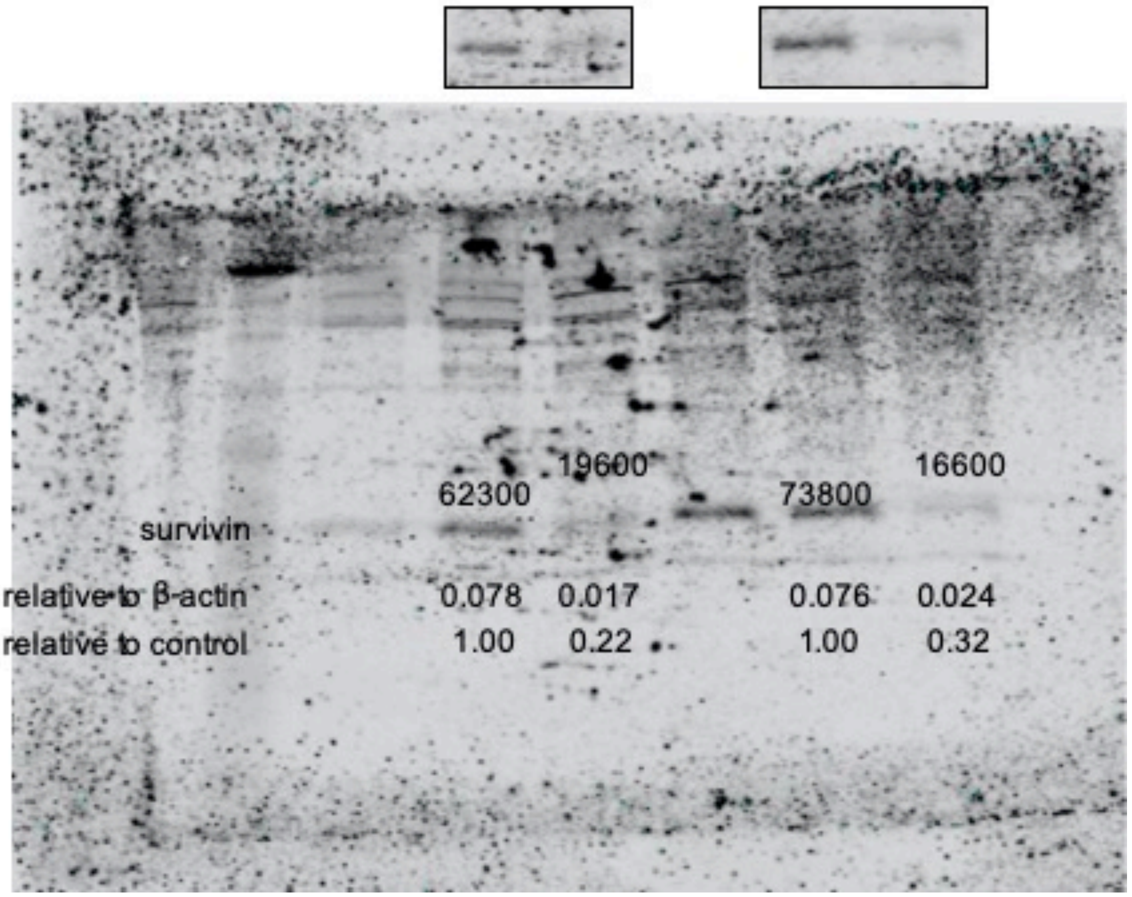

Figure S4
GAPDH

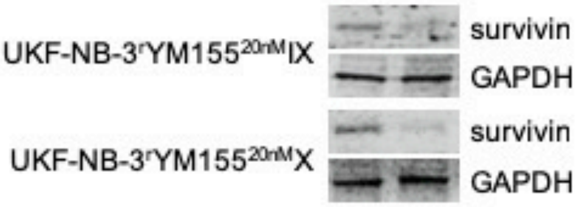

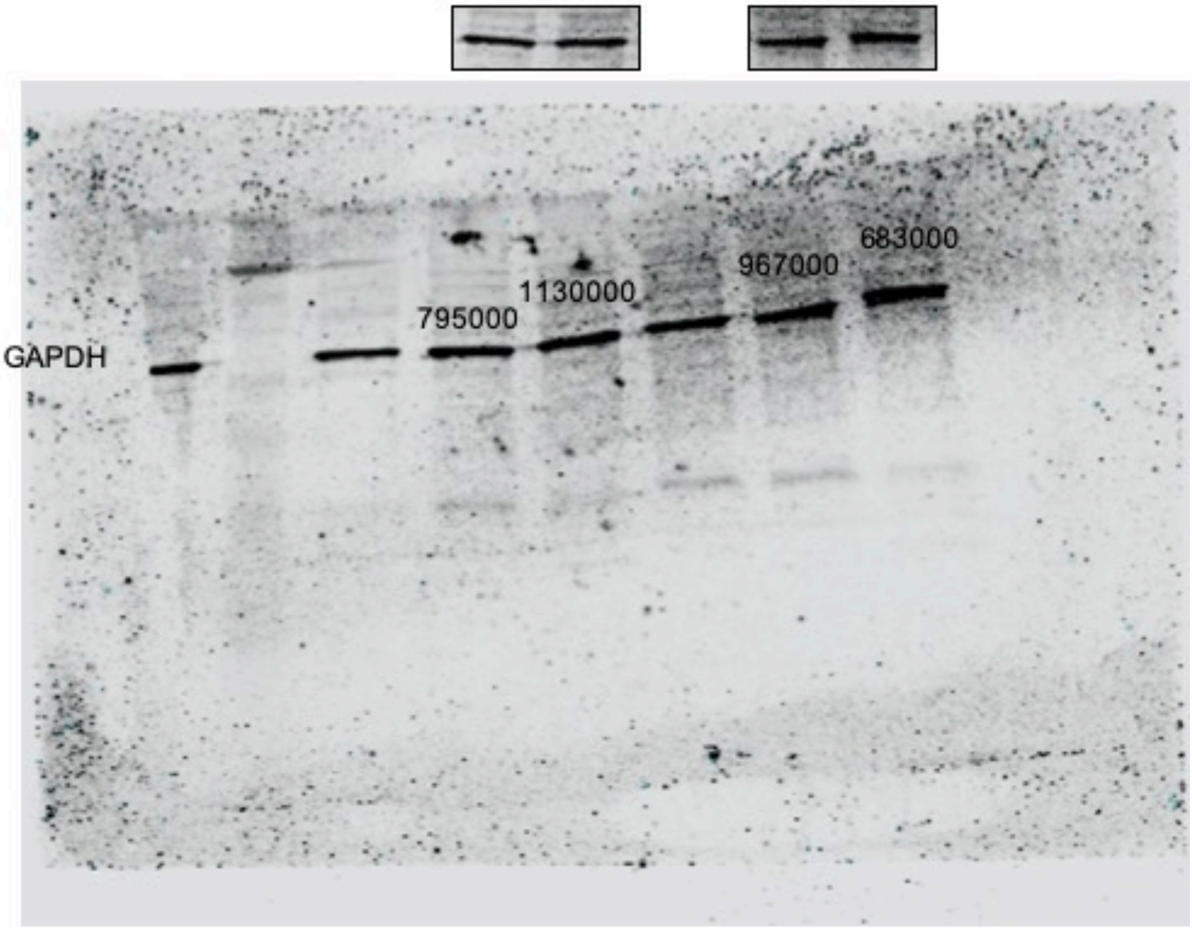

Figure S4. Representative Western blots indicating cellular levels of survivin in UKF-NB-3 and its YM155-adapted UKF-NB-3 sub-lines 24 h after transfection with non-targeting siRNA or siRNA directed against BIRC5/ survivin. Densitometric analysis was performed with QuantiOne (BioRad). Survivin levels were normalised to β-actin expression and values relative to control cells are displayed.

Figure S5
ABCB1

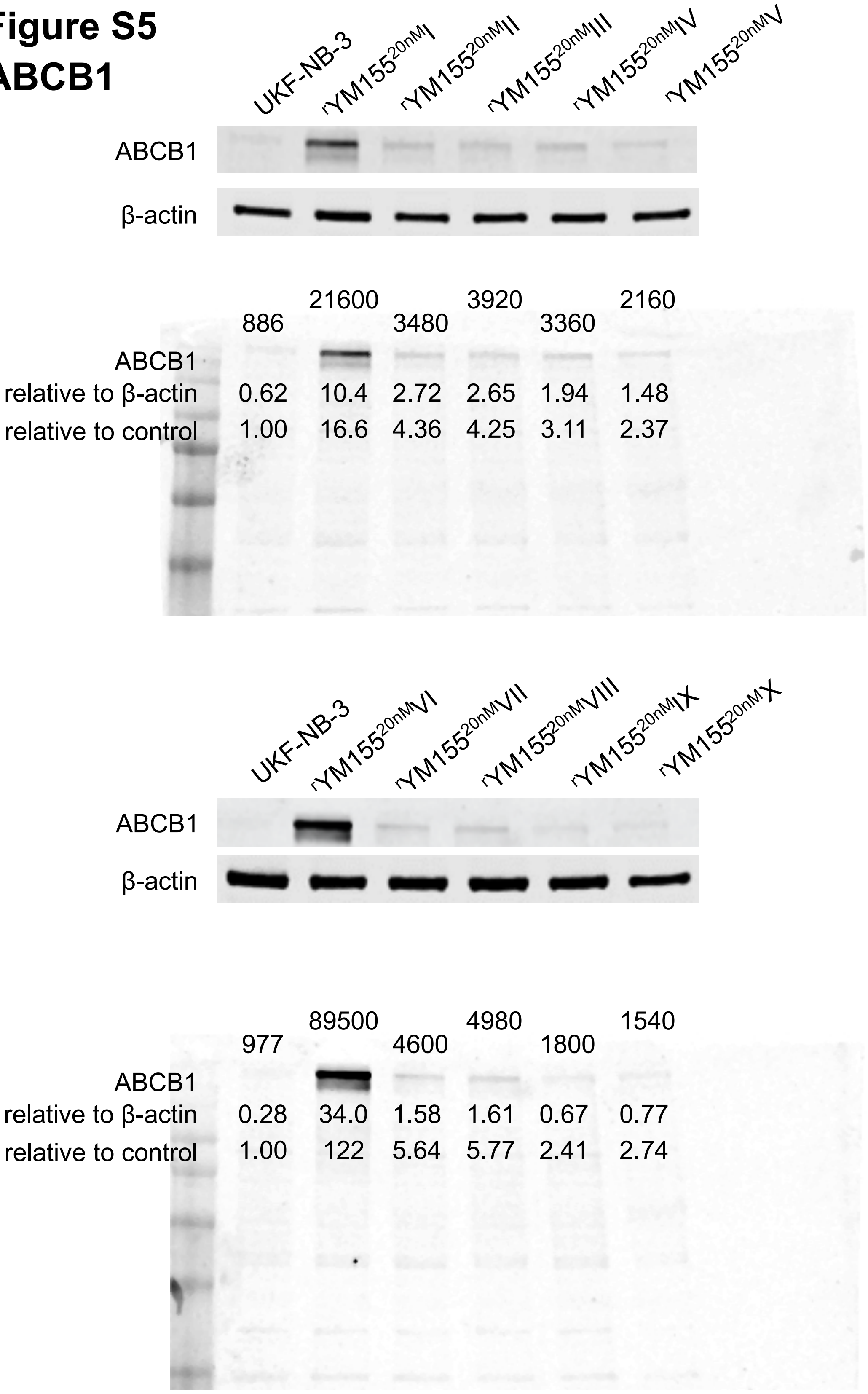

Figure S5
SLC35F2

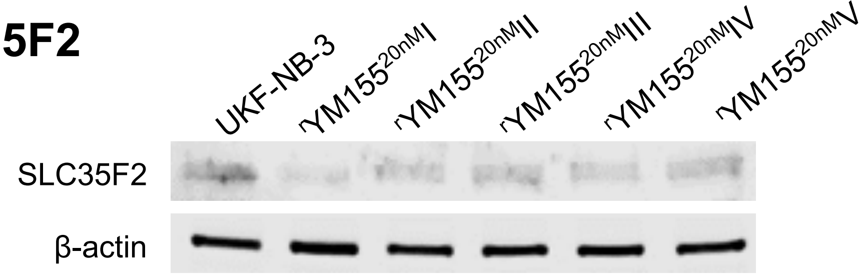

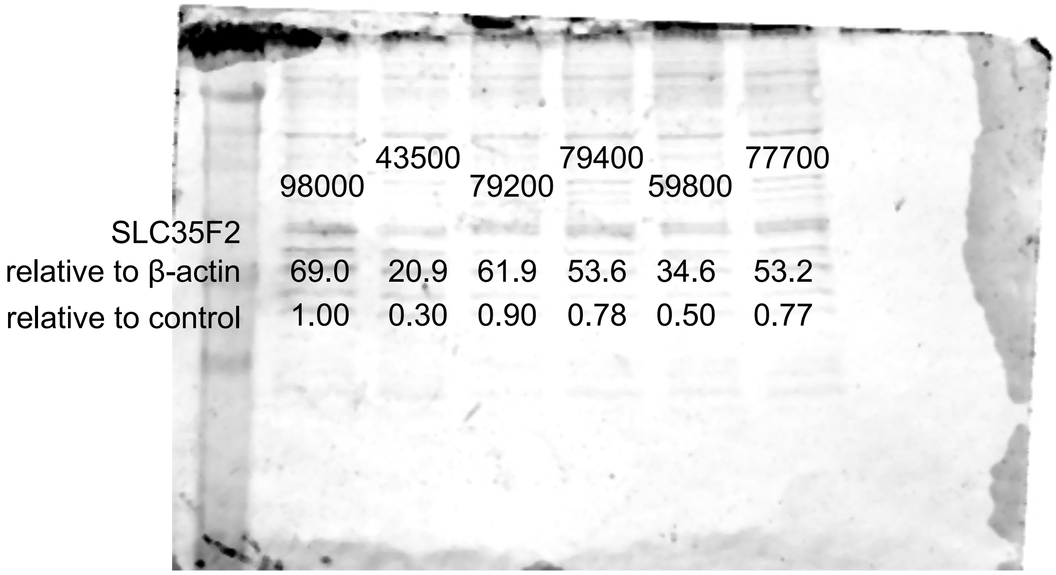

Figure S5

SLC35F2

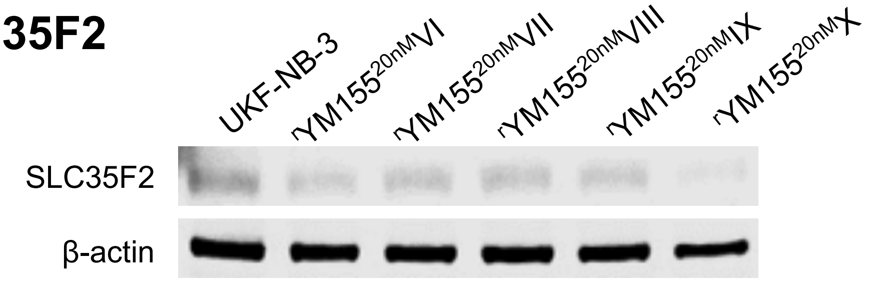

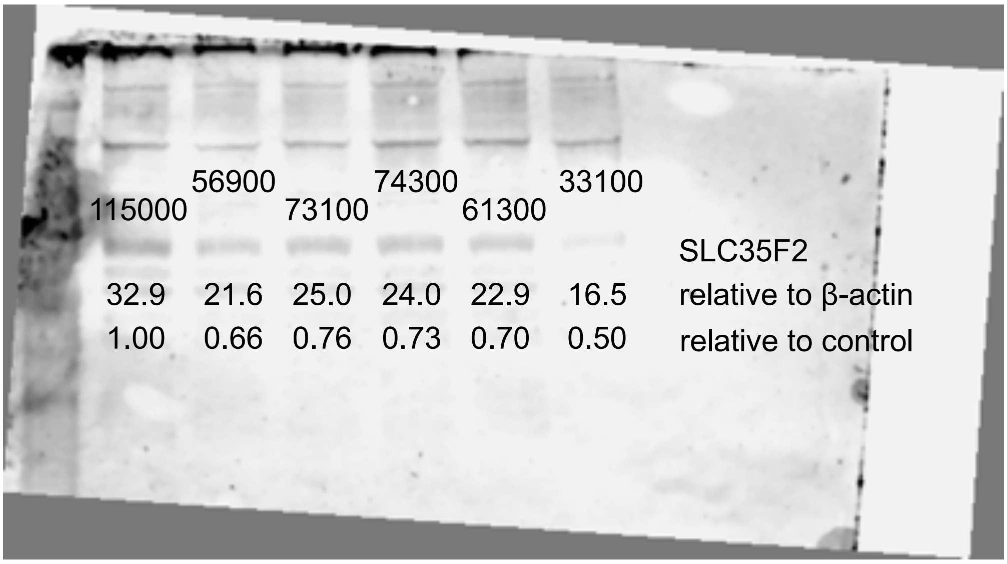

Figure S5

β-actin

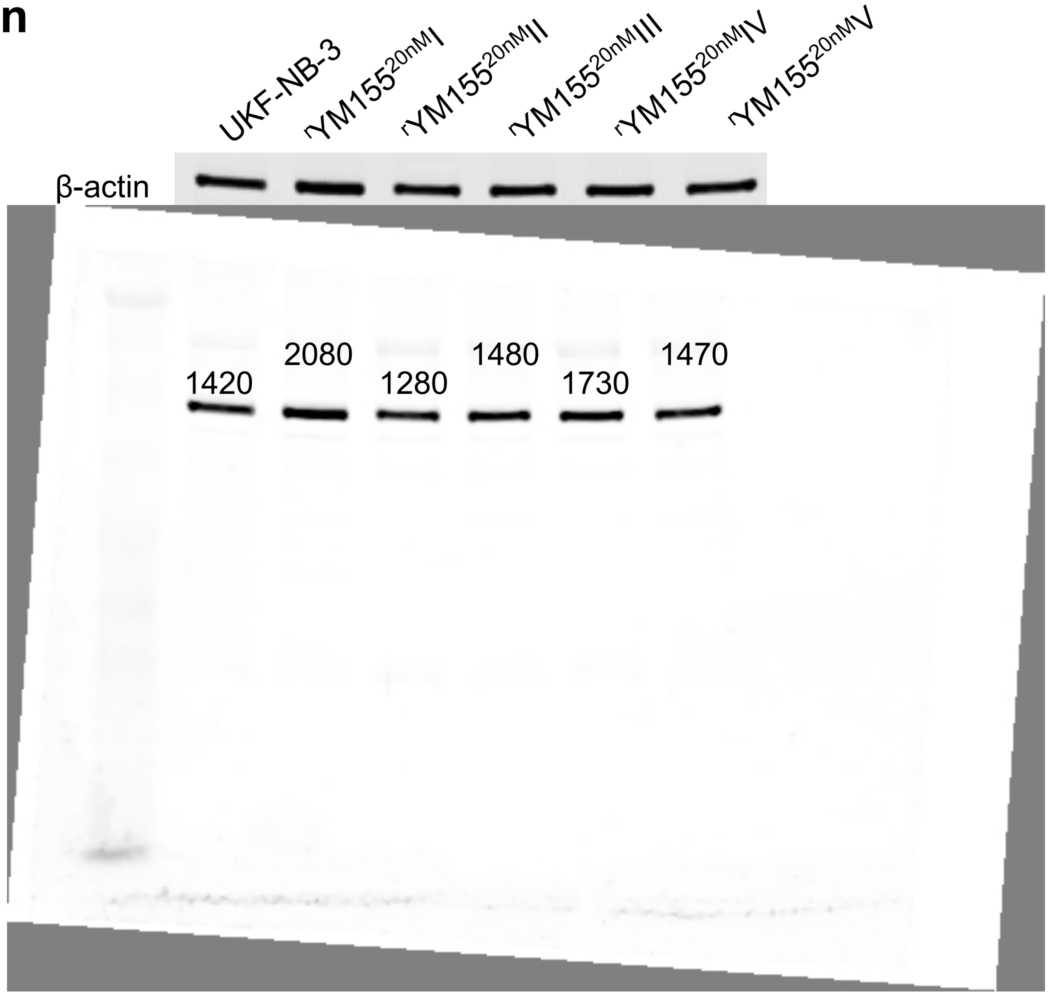

Figure S5
β-actin

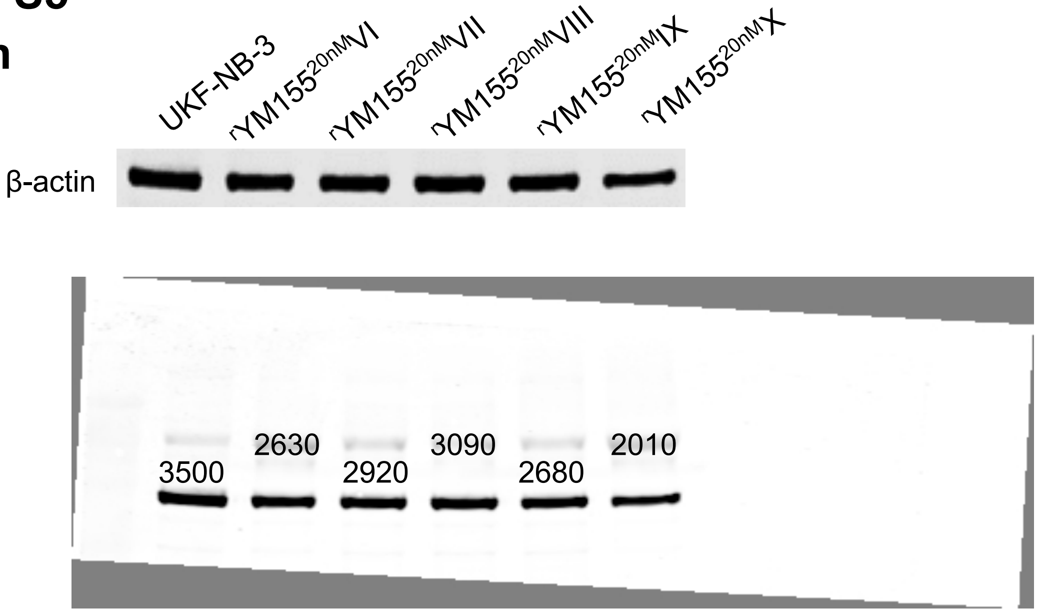

Figure S5. Representative Western blots indicating cellular levels of ABCB1 and SLC35F2 in UKF-NB-3 and YM155-adapted UKF-NB-3 sub-lines. Densitometric analysis was performed with QuantiOne (BioRad). ABCB1 and SLC35F2 levels were normalised to β-actin expression and values relative to control cells are displayed.

Figure S6

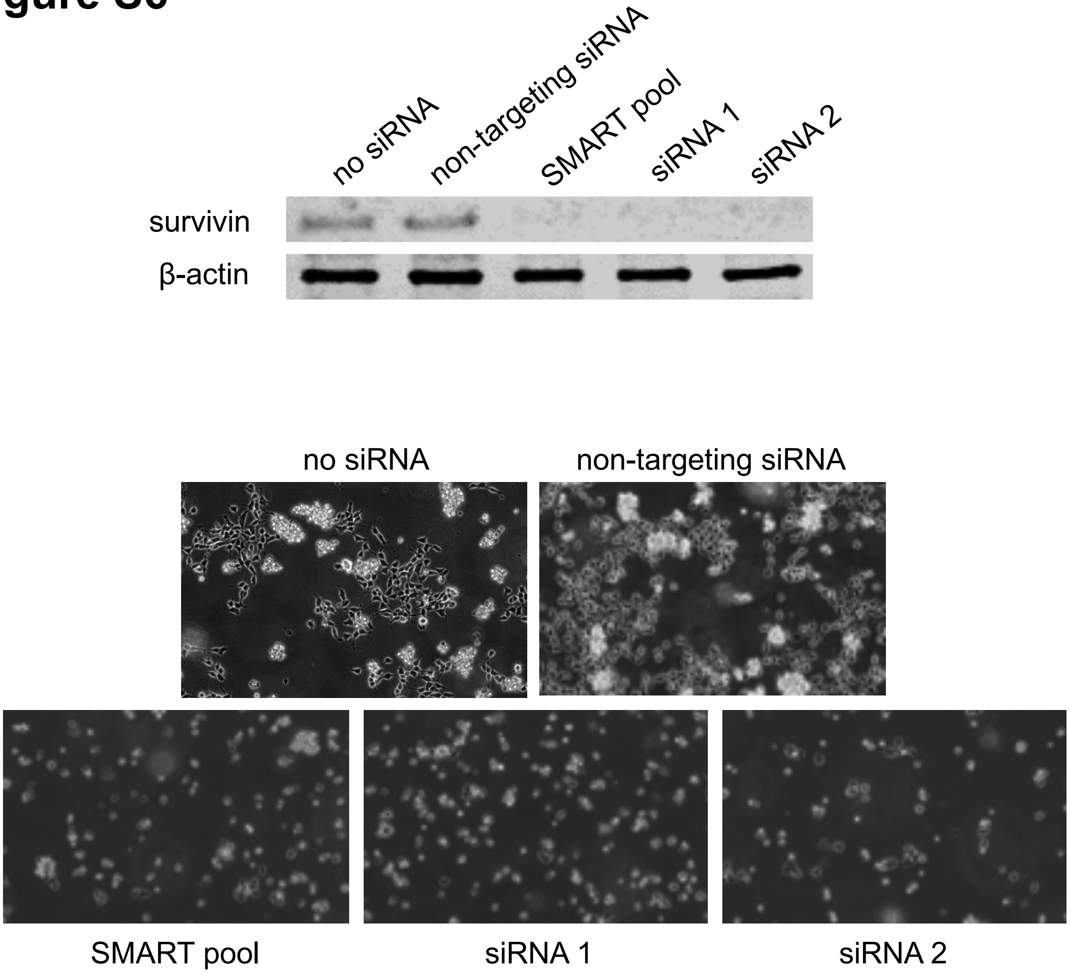

Figure S6. Western blots indicating cellular levels of survivin levels after transfection with the Dharmacon SMART pool (consisting of four siRNAs) and two individual siRNAs (siRNA 1, target sequence: GCAAAGGAAACCAACAAUA; siRNA 2, target sequence: GGAAAGGAGAUCAACAUUU) in UKF-NB-3 48h and representative images showing effects on cell viability..

Figure S6

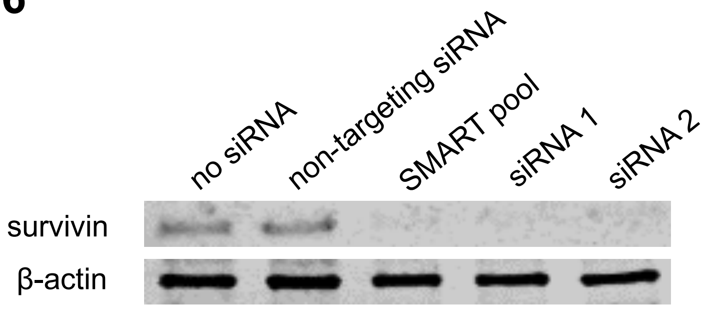

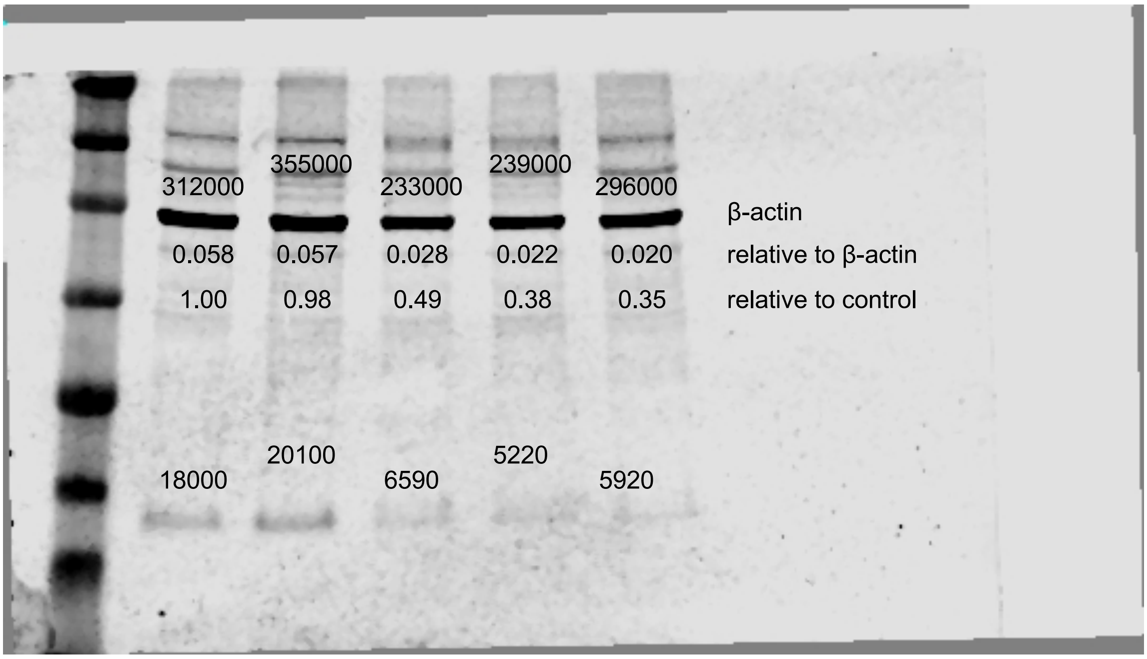

Figure S6. Original blots, densitometric analysis was performed with QuantiOne (BioRad). Survivin levels were normalised to β-actin expression and values relative to control cells are displayed.
